# Efficacy and Effectiveness of the Meningococcal Conjugate Group A Vaccine MenAfriVac^®^ in Preventing Recurrent Meningitis Epidemics in Sub-Saharan Africa

**DOI:** 10.3390/vaccines10040617

**Published:** 2022-04-14

**Authors:** Simonetta Viviani

**Affiliations:** Department of Molecular and Developmental Medicine, University of Siena, Via Aldo Moro 2, 53100 Siena, Italy; simonetta.viviani@unisi.it

**Keywords:** conjugate meningococcal vaccine, *Neisseria meningitidis* serogroup A, efficacy, effectiveness, meningitis epidemic, sub-Saharan Africa

## Abstract

For more than a century, epidemic meningococcal disease mainly caused by serogroup A *Neisseria meningitidis* has been an important public health problem in sub-Saharan Africa. To address this problem, an affordable meningococcal serogroup A conjugate vaccine, MenAfriVac^®^, was developed specifically for populations in the African meningitis belt countries. MenAfriVac^®^ was licensed based on safety and immunogenicity data for a population aged 1–29 years. In particular, the surrogate markers of clinical efficacy were considered to be the higher immunogenicity and the ability to prime immunological memory in infants and young children compared to a polysaccharide vaccine. Because of the magnitude of serogroup A meningitis epidemics and the high morbidity and mortality burden, the World Health Organization (WHO) recommended the MenAfriVac^®^ deployment strategy, starting with mass vaccination campaigns for 1–29-year-olds to rapidly interrupt serogroup A person-to-person transmission and establish herd protection, followed by routine immunization of infants and toddlers to sustain protection and prevent epidemics. After licensure and WHO prequalification of MenAfriVac^®^, campaigns began in December 2010 in Burkina Faso, Mali, and Niger. By the middle of 2011, it was clear that the vaccine was highly effective in preventing serogroup A carriage and disease. Post introduction meningitis surveillance revealed that serogroup A meningococcal disease had disappeared from all age groups, suggesting that robust herd immunity had been achieved.

## 1. Introduction

**Figure 1:**
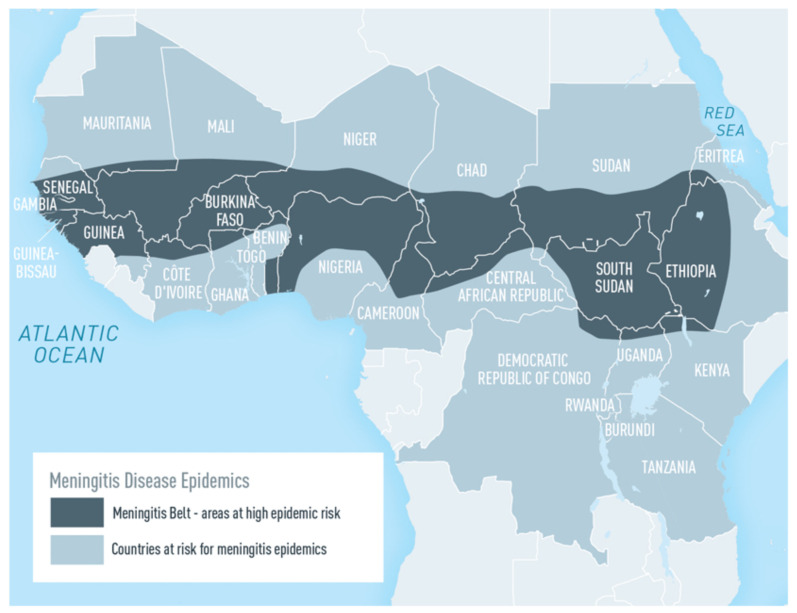
Areas with frequent epidemics of meningococcal meningitis. Disease data source: World Health Organization, International Travel and Health (Geneva, Switzerland, 2015). https://wwwnc.cdc.gov/travel/yellowbook/2020/travel-related-infectious-diseases/meningococcal-disease (accessed 23 March 2022).

For more than a century, and until a decade ago, recurrent epidemics of meningitis had been occurring in sub-Saharan Africa with well-known and described epidemiological patterns [1,2]. Every year from January to May during the “dry season,” when much of sub-Saharan Africa is dry, windy, and dusty, meningitis would arrive in epidemics that spread across several countries, starting from Senegal and The Gambia in the west to Ethiopia and Sudan in the east, along the so called “meningitis belt,” a term used for the first time by Lapeyssonie (Figure 1) [3].

Approximately every 10–15 years, meningococcal epidemics peaked with major proportions, such as 1996–1997, when the epidemic was estimated to have caused 250,000 cases of meningitis with more than 25,000 deaths [1,2,4]. Meningitis is one of the most feared diseases in the African continent and has a high burden of morbidity and mortality. Meningitis epidemics caused major social and economic disruptions of fragile national public health systems because of the severity of the illness that has a case-fatality rate of about 10%. Serious, long-lasting sequelae, such as deafness, mental retardation, and seizures, are reported in approximately 20% of survivors and represent an additional high economic burden on families and individuals [5]. The majority of meningitis epidemics were caused by *Neisseria meningitidis* serogroup A (NmA), which accounted for 80–85% of isolates and occurred in persons between 1 and 29 years of age [6,7].

Since the 1970s and 1980s, capsular polysaccharide vaccines inducing group-specific functional antibodies have been licensed and used on the basis of Gotschlich’s classical studies [8]. These vaccines were shown to decrease disease caused by NmA, *Neisseria meningitidis* serogroup C (NmC), *Neisseria meningitidis* serogroup Y (NmY), and *Neisseria meningitidis* serogroup W135 (NmW) [8]. In the 1970s, a serogroup A meningococcal polysaccharide vaccine tested in field studies in Africa was found to be effective in 1–29-year-olds for reducing meningococcal disease [9,10,11]. Group A + C polysaccharide vaccines were used for more than 30 years to control meningitis epidemics in sub-Saharan Africa during reactive vaccination campaigns, as recommended by the World Health Organization (WHO) for management and control of meningococcal disease [12]. Although polysaccharide vaccines reduced cases of meningitis and were able to curb epidemics, they had no impact on preventing them, and epidemic waves persisted in sub-Saharan Africa despite the administration of millions of vaccine doses [13,14]. Indeed, polysaccharide vaccines are B-cell-dependent antigens, are not immunogenic in infants and young children, induce antibodies that last for 3–5 years only and, most important, they are not able to induce immunological memory, so have little or no effect on nasopharyngeal carriage and in preventing person-to-person transmission [13,14]. In the 1980s, the development of polysaccharide conjugate vaccines against *Haemophilus influenzae* serotype B (Hib) showed that polysaccharide antigens coupled to proteins such as tetanus toxoid, diphtheria toxoid, and CRM197, a genetically detoxified form of diphtheria toxoid, become T-cell-dependent antigens for a more potent response than polysaccharides alone [15]. Conjugate polysaccharide antigens are immunogenic in infants as young as a few weeks old, and are able to prime immunological memory, thereby reducing nasopharyngeal carriage and preventing human-to-human transmission. Several Hib conjugate vaccines developed in the 1990s were included in infants’ vaccination programs in the USA and in some European countries, leading to a drastic reduction in Hib invasive disease and nasopharyngeal carriage, and produced a strong herd immunity effect [16,17]. In 1998, on the basis of this major public health achievement, WHO issued the recommendation for introducing the Hib conjugate vaccine for universal vaccination of infants in the Expanded Program on Immunization (EPI) [18]. In the 1990s, attempts to develop meningococcal conjugate vaccines were made, and clinical trials were performed in Niger [19] and in The Gambia [20] with two different serogroup AC conjugate vaccines. Unfortunately, these trials were never followed by further development, and those vaccines were never made available. After the dramatic 1996–1997 NmA meningitis epidemic in Africa, it became evident that new improved meningococcal vaccines were urgently needed. As reported by Aguado and colleagues [21], at the end of the 1990s, an intense initiative was undertaken by WHO to urge vaccine manufacturers to develop meningococcal conjugate vaccines containing serogroup A or AC [21,22] to be used in Africa. While the initiative was unsuccessful, meningococcal conjugate group C vaccines were licensed in 1999 in Europe and first used in mass vaccination campaigns in the United Kingdom (UK) to prevent meningitis outbreaks caused by NmC [21]. No other meningococcal conjugate vaccines were licensed until 2005, when the first meningococcal conjugate polyvalent vaccine Menactra^®^ containing polysaccharides from NmA, NmC, NmY, and NmW conjugated to the diphtheria toxoid protein was licensed in the USA [23].

## 2. Implementation of the Meningitis Vaccine Project (MVP), the Organization, the Scientific and Technical Partnerships, and Collaboration

In 2001, a structured proposal including a vaccine development plan assembled by WHO and PATH (Program for Appropriate Technology on Health), a Seattle-based non-governmental organization with experience in developing appropriate technology for poor settings, with support from several partners including the Centers for Disease Control and Prevention (CDC), was awarded a grant by the Bill & Melinda Gates Foundation (BMGF). The Meningitis Vaccine Project (MVP) was born as a partnership between WHO and PATH with core funds from BMGF with the scope to develop and license a meningococcal conjugate vaccine to prevent meningitis epidemics in sub-Saharan Africa [21,24]. At the end of 2009, the meningococcal serogroup A conjugate vaccine, MenAfriVac^®^, was licensed by DCGI (Drug Controller General of India) for manufacture by Serum Institute of India Ltd., Pune (SIIL), and in June 2010, it was prequalified by WHO. Mass vaccination of the 1- to 29-year-old population started in late 2010 in Burkina Faso, Mali, and Niger. In 2014, WHO recommended that vaccination with MenAfriVac^®^ be included in the infant vaccination programs of meningitis belt countries.

The various steps for the implementation of the MVP, the organization, the scientific and technical partnerships, and collaboration, as well as the strategy to produce and fund an affordable vaccine, have been extensively described elsewhere [21,24,25,26,27]. In this article, the technical and scientific strategy pursued to develop a vaccine with high efficacy and effectiveness for preventing meningitis epidemics caused by *Neisseria meningitidis* group A will be described and reviewed.

## 3. Technical and Scientific Strategy Pursued to Develop a Vaccine with High Efficacy and Effectiveness for Preventing Meningitis Epidemics Caused by Neisseria Meningitidis Group A

### 3.1. MenAfriVac^®^ Efficacy

Since the inception of the MVP in 2001, it was clear that to eliminate recurrent epidemics of meningitis, a highly efficacious conjugate vaccine was needed that could be administered in a single dose to 1- to 29-year-olds residing in the meningitis belt countries [26]. The technology to develop conjugate vaccines has been in place since the early 1990s and was successfully applied to develop and license Hib and NmC conjugate vaccines [24]. Conjugate vaccines differ from each other mainly on the basis of the size of the polysaccharide and the conjugation protein used; for the latter, tetanus toxoid, diphtheria toxoid, and CRM197, a genetically detoxified form of diphtheria toxoid, are among the most commonly used.

Although some studies suggested that the unsized Hib polysaccharide conjugated to tetanus toxoid protein was able to induce the highest level of high-avidity antibodies in infants [28], no data were available on the optimal size of the polysaccharide or the type of conjugation protein for a group A polysaccharide conjugate [29]. In addition, most of the technological platforms were protected by patents that blocked vaccine development or use [27]. Finally, a conjugation technology developed at the US National Institutes of Health (NIH) was identified, transferred, and further developed at SIIL, the vaccine manufacturer [27,29]. The optimal vaccine candidate was studied in terms of molecular size, molecular weight, degree of absorption of the conjugate to aluminium phosphate, and effect of the adjuvant on immunogenicity in animal models [27,29]. The final vaccine formulation to be tested in humans comprised 10 µg of group A polysaccharide conjugated to tetanus toxoid with aluminium phosphate as adjuvant [25,29]. The demonstration of the efficacy of MenAfriVac^®^ in field trials was deemed unfeasible due to the unpredictability of the geographical onset and spread of meningitis epidemics [22,26,27]. Therefore, for the purpose of vaccine licensure, a clinical development plan was designed to assess—besides safety—the immunogenicity and ability of MenAfriVac^®^ to induce immunological memory when compared to an unconjugated polysaccharide vaccine. The ability to prime immunological memory in infants and young children as assessed in clinical studies was considered a marker of vaccine efficacy since unconjugated polysaccharide vaccines are poorly, if at all, immunogenic in children younger than 2 years and cannot induce immunological memory [30,31,32]. On this basis, and without pre-licensure for field efficacy trials, NmC conjugate vaccines were licensed in the UK and deployed in a mass vaccination campaign for children and adolescents to successfully prevent outbreaks of NmC, which had been occurring for decades in the young with a high mortality and morbidity burden [33]. Immunogenicity was evaluated using Serum Bactericidal Activity (SBA), a well-established and standardized serological assay that assesses the complement-dependent bactericidal activity of antibodies in sera against serotype-specific meningococcus isolates, and was deemed to be a marker of clinical protection [8,34]. NmC conjugate vaccines were very effective in the UK, where they induced a rapid and significant reduction in meningococcal invasive disease and carriage thanks to [35,36] the indirect effect of herd immunity in the unvaccinated population [37] and long-lasting vaccine effectiveness [38]. Two types of complement can be used in the SBA assay, rabbit complement and human complement, which is deemed to exclusively bind high-avidity antibodies. Serological correlates for protection against invasive meningococcal disease were established in the UK by post-licensure studies in toddlers. More than 90% of toddlers, who after vaccination had rSBA antibody titers between 8 and 64 (using rabbit complement) and with a hSBA titer > 4 (using human complement), were protected against invasive NmC disease. A 4-fold increase in rSBA titers after vaccination (compared to baseline titers) was also found to correlate with protection [39,40]. The MenAfriVac^®^ clinical development plan was based on the NmC vaccine development experience and existing guidelines. The MenAfriVac^®^ Phase I clinical study was performed on healthy adults in India. Subjects vaccinated with one dose of MenAfriVac^®^ had a higher proportion with a 4-fold increase in rSBA compared to baseline titers, as well as higher rSBA titers when expressed as geometric mean titers (GMTs), than subjects vaccinated with a licensed polysaccharide group A + C vaccine [41]. Although these data provided an indication of the better performance of MenAfriVac^®^ over a polysaccharide vaccine in adults, the subsequent clinical study performed in 12- to 24-month-old children in Mali and The Gambia, two countries located in the meningitis belt, by Sow and colleagues provided unequivocal evidence. MenAfriVac^®^ was a potent conjugate vaccine with superior immunogenicity with respect to a quadrivalent ACYW135 polysaccharide vaccine in terms of a 4-fold increase in rSBA, rSBA geometric mean titers (GMTs), and the proportion of subjects with a rSBA titer ≥ 128 after vaccination [42]. The 4-fold increase criterion was very discriminative, as found in the UK [39,40], as were GMTs, however rSBA dilutions below 1:128 were not very discriminative in this population due to high rSBA baseline titers likely induced by intense circulation of cross-reactive bacteria [43,44]. The study was, in fact, designed to evaluate cross reactivity with bacteria other than meningococci in subjects vaccinated with a Hib conjugate vaccine and boosted 6–8 months later with either the Hib conjugate vaccine, 1/5 dose of polysaccharide ACWY, or MenAfriVac^®^. The study design allowed the authors to conclude that a single dose of MenAfriVac^®^ in 12–24 month-old children showed superior immunogenicity when compared to the polysaccharide NmA vaccine, and that the vaccine was able to induce immunological memory when boosting the same subjects 6–8 months later with either 1/5 dose of polysaccharide ACWY vaccine or MenAfriVac^®^ itself [42]. These data were considered to represent surrogate proof for the clinical efficacy of MenAfriVac^®^ since they were generated in young children in meningitis belt countries where only meningococcal A + C polysaccharide vaccines were used for reactive vaccination campaigns and considered the best available option in those settings [45]. Other clinical studies were performed in other meningitis belt countries, such as Senegal, Mali and The Gambia, and also in India, to support the safety and immunogenicity of MenAfriVac^®^ compared to meningococcal polysaccharide vaccines in the 2- to 29-year-old population and obtain vaccine licensure in India in 2009 and WHO prequalification in 2010 [42,46]. In 2011, WHO recommended vaccination with one dose of MenAfriVac^®^ for mass immunization of the 1–29-year-old population residing in meningitis belt countries [46]. After 2010, as preventive vaccination campaigns for 1- to 29-year-olds were taking place in meningitis belt countries, new clinical studies were performed in infants in Mali and in Northern Ghana to support the introduction of MenAfriVac^®^ to national infant immunization programs [47,48]. The immunogenicity results from these studies confirmed the data generated by the pivotal phase II study in 12- to 24-month-olds [42] and further supported the efficacy of MenAfriVac^®^ for preventing invasive meningococcal disease caused by NmA in infants and young children [48]. On this basis, in 2015 WHO recommended MenAfriVac^®^ vaccination for infants living in meningitis belt countries with either one dose between 9 and 15 months of age, or two doses given at least 8 weeks apart starting from 3 months of age [48].

### 3.2. MenAfriVac^®^ Effectivness

**Figure 2:**
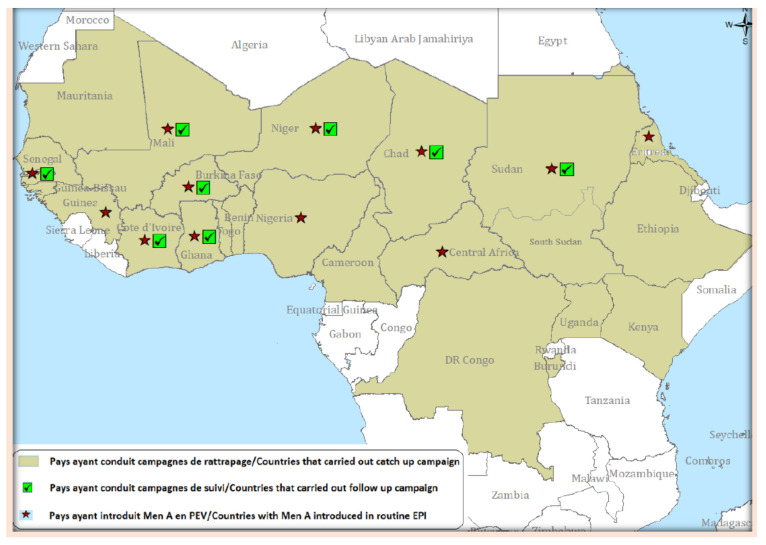
MenAfriVac^®^ introduction in the meningitis belt countries, 2010–2021. From WHO/IST-WA Meningitis Weekly Bulletin, 2021 (https://cdn.who.int/media/docs/default-source/2021-dha-docs/bulletin-meningite_s26_2021.pdf?sfvrsn=3512cc8e_1&download=true) (accessed 22 March 2022).

Since the goal of the MVP was to eliminate group A recurrent meningococcal epidemics from the meningitis belt countries, along with defining and pursuing a strategy for developing, testing, and licensing MenAfriVac^®^, an enhanced meningitis surveillance was established in 2003 at WHO’s Multi-Disease Surveillance Centre in Ouagadougou, Burkina Faso [27,49,50]. A dedicated team created a specific meningitis surveillance network for the meningitis belt countries, including case definitions for suspected meningitis, confirmed meningitis, outbreak and epidemic alerts, strengthening of local reference laboratories and coordination with international reference laboratories, data collection, data management, and publication of epidemiological periodic bulletins, with a weekly frequency during the meningitis season (January–June) and a monthly frequency during the non-meningitis season [27,49,50]. Initially, in 2003, eight countries (Benin, Burkina Faso, Chad, Ghana, Mali, Niger, Nigeria and Togo) contributed to this enhanced surveillance network. The number of reporting countries increased to 13 in 2004, and introduction of MenAfriVac^®^ by mass campaigns for 1- to 29-year-olds across the meningitis belt countries initiated in 2010 [47] further drove up country participation in the meningitis enhanced surveillance network. By 2013, 19 countries (Benin, Burkina Faso, Cameroon, Central African Republic, Chad, Democratic Republic of Congo, Ethiopia, Ghana, Guinea, Côte d’Ivoire, Mali, Mauritania, Niger, Nigeria, Senegal, South Sudan, Sudan, The Gambia and Togo) reported to the enhanced surveillance network [50]. An evaluation of the meningitis surveillance in Mali and Burkina Faso performed before the introduction of MenAfriVac^®^ [51] found that, although some need for improvement was identified and actions recommended, the ability of the system to detect cases of meningitis in a timely manner, provide laboratory confirmation, and disseminate results for action had greatly improved over the years. In addition, the meningitis surveillance network was deemed to be sufficiently sensitive (case detection, district alert outbreaks, and alert epidemic threshold) and specific (substantial proportion of meningitis cases with laboratory confirmation) for identifying meningitis outbreaks, if any, in the ten countries under study (Benin, Burkina Faso, Chad, Democratic Republic of Congo, Ghana, Côte d’Ivoire, Mali, Niger, Nigeria, Togo), and thus, for evaluating MenAfriVac^®^ effectiveness in preventing meningitis epidemics caused by NmA [51]. A 10-year analysis showed that the highest meningitis peak occurred in 2009 (86,714 cases), corresponding to a major epidemic in Niger and Nigeria, and the second-highest peak in 2007 (45,195 cases) was due to an epidemic in Burkina Faso. Case fatality ranged from 13.7% in 2004 and 2005 to 10% in subsequent years apart from 2009, the year of the highest incidence, when fatality fell to 5.9%. The seasonal patterns with peaks in the first five months of the year (January–June) were confirmed. Until 2010, before MenAfriVac^®^ introduction, the enhanced surveillance network data confirmed that NmA was the main cause of meningitis [47,50] despite local outbreaks of NmW in Burkina Faso in 2006 and 2009–2010, and of NmX in Niger in 2006 [47,50,52]. Since December 2010, mass vaccination campaigns have been rolled out in the meningitis belt countries according to the district prioritization tool (DPT) [53] used to rank countries for planning mass vaccination campaigns, as well as for future catch-up campaigns for unvaccinated young cohorts, and introduction into routine immunization programs. The DPT was developed for sequential country introductions based on epidemic risk, disease burden, and the ability of countries to conduct vaccination campaigns. Using this tool, countries were ranked into the following five risk categories: Group 1, countries with high epidemic risk and high disease burden (Burkina Faso, Chad, Ethiopia, Mali, Niger, Nigeria and Sudan); Group 2, countries with high epidemic risk but low disease burden (Benin, Cameroon and Ghana); Group 3, countries with low epidemic risk but high disease burden (Democratic Republic of the Congo and South Sudan); Group 4, countries with an intermediate epidemic risk and disease burden (Côte d’Ivoire, Guinea, Senegal, Togo and Uganda); Group 5, countries with low epidemic risk and low disease burden (Burundi, Central African Republic, Eritrea, Gambia, Guinea-Bissau, Kenya, Mauritania, Rwanda and United Republic of Tanzania) [53]. Nationwide mass vaccination of 1–29-year-olds with a single dose of MenAfriVac^®^ started in Burkina Faso in December 2010, was completed in Mali and Niger in 2011 and in Chad in 2012. In subsequent years up to 2021, most of the meningitis belt countries had completed mass vaccination of 1- to 29-year-olds either with nationwide coverage, or coverage in areas identified as at high risk of meningitis according the DPT (Figure 2) [54,55].

Since the introduction of MenAfriVac^®^, the overall incidence of meningitis in African meningitis belt countries has decreased steadily along with the risk of meningitis epidemics [54,55,56,57,58,59]. NmA cases have disappeared completely in most countries, with sporadic cases reported in unvaccinated individuals in Burkina Faso, Cameroon, Chad, Guinea, Niger, Nigeria, Senegal, Guinea, and Nigeria. A single vaccine failure has been documented in Burkina Faso [55,56,57,58,59,60]. After nationwide vaccination campaigns with MenAfriVac^®^, Burkina Faso reported a 71% decline in the risk of suspected meningitis, a 64% decline in the risk of fatal meningitis, and a >99% decline in the risk of confirmed NmA meningitis. A rapid herd immunity effect was shown to be statistically significant, leading to a risk reduction for meningitis in the unvaccinated population younger than 1 year and older than 30 years that were not eligible for vaccination [56]. Similar data were found in Chad during phased nationwide vaccination with MenAfriVac^®^, with a 94% reduction in the incidence of suspected meningitis in vaccinated versus unvaccinated districts, and a 98% decrease in asymptomatic NmA carriage prevalence 4–6 months after mass vaccination vs. the pre-vaccination era [57]. A study in nine countries (Benin, Burkina Faso, Chad, Côte d’Ivoire, Ghana, Mali, Niger, Nigeria, and Togo) that was carried out between 2010 and 2015 during the mass vaccination campaigns showed a 58% decline in the incidence of suspected meningitis, a >99% decline in the incidence of laboratory confirmed NmA meningitis, a 60% decline in the risk of epidemics, and an increase in the incidence of non-NmA meningitis [58]. In countries participating in the MenAfriNet Consortium [59] and considered at very high risk for meningitis (Burkina Faso, Chad, Mali, Niger, and Togo), between 2015 and 2017, it was found that bacterial meningitis epidemiology varied widely by country, with NmC and NmW causing several outbreaks and NmX increasing although not associated with outbreaks. Only five cases of NmA meningitis were identified in Burkina Faso in 2015, of which one was the first documented vaccine failure [60]. Carriage studies performed in Burkina Faso and Chad showed the near disappearance of serogroup A carriage in the vaccinated and unvaccinated population, showing again the herd immunity effect generated by vaccination with MenAfriVac^®^ and its effectiveness in preventing meningitis epidemics [57,61,62].

## 4. Conclusions and Future Direction

Continuous systematic surveillance in the meningitis belt countries shows that meningitis epidemics caused by NmA have not been reported for more than 10 years, and that serogroup A is rarely isolated [63,64,65] in confirmed cases since more countries have conducted vaccination of the 1–29-year-old population, performed catch-up campaigns for the 1–5-year-old population, or introduced vaccination in the EPI with MenAfriVac^®^ [64,65]. The demonstration of its higher immunogenicity and the ability to prime immunological memory in infants and young children when compared to a polysaccharide vaccine was considered a surrogate marker for the clinical efficacy of the conjugate NmA vaccine MenAfriVac^®^, specifically developed for the elimination of recurrent meningitis epidemics due to NmA in sub-Saharan Africa. After MenAfriVac^®^ introduction and roll-out in the meningitis belt countries, epidemics of NmA have disappeared and NmA is rarely isolated. The amazing effectiveness of MenAfriVac^®^ is due to, on one hand, its properties in terms of high immunogenicity and the ability to prime immunological memory, and on the other hand, the crucial role played by a vaccination strategy that initially included campaigns for the 1- to 29-year-old high-risk population conducted over a short period of time. These two factors allowed rapid interruption of person-to-person transmission and produced a striking herd immunity effect with protection extended to the unvaccinated population. The subsequent catch-up campaigns for 1 to 5 years old and inclusion in infant vaccination programs contributed to further reductions in unvaccinated cohorts and the maintenance of herd immunity. However, severe outbreaks of meningitis with high fatality rates caused by NmC and NmW have been reported between 2011 and 2019 [63,64] in Nigeria, Niger, Togo, and Chad, and with mixed circulation of serogroups X and W in Burkina Faso. Several reactive vaccination campaigns with ACW or ACWY polysaccharide vaccines have been conducted to control the spread of the outbreaks without much effect in preventing the recurrence of such outbreaks [63,64]. In addition, no polysaccharide vaccine is available for serogroup X.

Since a conjugate polysaccharide polyvalent meningitis vaccine against Nm serogroups ACYWX has been developed [66], and has been shown to be safe and highly immunogenic in toddlers in Mali [67,68], the potential to definitively eliminate severe outbreaks of meningitis caused by NmC, NmW, and NmX can become a reality.

The new conjugate polyvalent meningitis vaccine against NmA, NmC, NmY, NmW, and NmX has been shown to be highly immunogenic and is able to prime immunological memory in toddlers. Since outbreaks of meningitis caused by non-NmA serotypes contained in this new vaccine continue to occur in the meningitis belt countries, an efficient vaccination strategy should be implemented to prevent these outbreaks and, at the same time, sustain the effectiveness achieved by MenAfriVac^®^ in preventing meningitis epidemics of NmA [69,70].

## Data Availability

Not applicable.
